# Quantitative Gait Analysis Using a Motorized Treadmill System Sensitively Detects Motor Abnormalities in Mice Expressing ATPase Defective Spastin

**DOI:** 10.1371/journal.pone.0152413

**Published:** 2016-03-28

**Authors:** James W. Connell, Rachel Allison, Evan Reid

**Affiliations:** Department of Medical Genetics and Cambridge Institute for Medical Research, University of Cambridge, Cambridge, United Kingdom; University of Sheffield, UNITED KINGDOM

## Abstract

The hereditary spastic paraplegias (HSPs) are genetic conditions in which there is progressive axonal degeneration in the corticospinal tract. Autosomal dominant mutations, including nonsense, frameshift and missense changes, in the gene encoding the microtubule severing ATPase spastin are the most common cause of HSP in North America and northern Europe. In this study we report quantitative gait analysis using a motorized treadmill system, carried out on mice knocked-in for a disease-associated mutation affecting a critical residue in the Walker A motif of the spastin ATPase domain. At 4 months and at one year of age homozygous mutant mice had a number of abnormal gait parameters, including in stride length and stride duration, compared to heterozygous and wild-type littermates. Gait parameters in heterozygous animals did not differ from wild-type littermates. We conclude that quantitative gait analysis using the DigiGait system sensitively detects motor abnormalities in a hereditary spastic paraplegia model, and would be a useful method for analyzing the effects of pharmacological treatments for HSP.

## Introduction

Hereditary spastic paraplegias (HSP) are genetically determined subtypes of motor neuron disease that are characterized by dying-back degeneration of the axons of the corticospinal tract [1–5]. In humans, they cause a progressive spastic gait abnormality that can begin from childhood to late adult life. No treatments for the HSPs are available presently, but potential therapeutic approaches, such as inhibition of BMP signaling or manipulation of microtubule dynamics, have been suggested [4, 6]. Thus there is a need to develop appropriate model systems and reliable methodologies with which to test therapeutic effects.

Mutations in SPAST/SPG4, which encodes the microtubule severing ATPase spastin, are the most frequent cause of HSP [7]. In North America and northern Europe, mutations in this gene account for up to 40% of autosomal dominant uncomplicated HSP and around 10% of sporadic cases [8–11]. SPAST mutations are also found in other geographical regions and ethnic populations [12–15]. The mutational spectrum associated with spastin-HSP is broad; frameshift, nonsense and splice site mutations, as well as larger genomic re-arrangements causing whole-exon deletions, are frequent [8, 9, 11]. Missense mutations are also common and these cluster in the ATPase domain [4]. Although other mechanisms have been suggested [16], this broad mutational spectrum suggests a haplo-insufficiency disease mechanism. The description of pathological genomic deletions involving the entire coding region of the gene, making protein expression from the affected allele impossible, strongly supports this idea [11].

To sever microtubules, current models suggest that the spastin ATPase domains hexamerise into a ring structure with a central pore [17–19]. ATP hydrolysis is then coupled to microtubule severing, by a mechanism involving the interaction between central pore residues and the C-terminal tail of tubulin [17, 18]. The known ATPase domain missense mutations appear to fall into several molecular pathogenic groups; they may block ATP binding or hydrolysis, prevent hexamerisation by interfering with ATPase domain protomer-protomer interactions or affect the interaction between pore residues and tubulin [17, 18].

Two spastin mouse models have been published. Tarrade *et al*. used a gene targeting approach to generate mice deleted for exons 5–7 of spastin [20]. The deletion caused a new splicing event linking exons 4 and 8, resulting in a frameshift that introduced a stop codon shortly after the exon 4–8 junction. In homozygous mice this caused complete absence of the spastin protein, probably because the truncated transcript is subject to nonsense-mediated decay. In cultured primary neurons from homozygous mutant mice, and to a lesser degree, heterozygous mice, this caused an axonal swelling phenotype that predominantly involved the distal axon. Homozygous mutant mice developed a mild, late onset motor defect. At 22 months of age footprint analysis of spontaneously walking homozygous mutant mice demonstrated a slightly increased step length and decreased uniformity of step length and step alternation compared to controls. In addition, between 15 and 24 months old, aerobic motor capacity declined faster in the homozygous mutants than in control mice. Kasher *et al*. also described a spastin loss-of-function model, in this case involving a mutation in the exon 7 splice donor site that caused exon 7 to be skipped from the transcript [21]. Again, homozygous mutant mice had no detectable spastin protein. These mice showed very similar axonal swellings to those described previously. The mice developed a mild gait abnormality detected during spontaneous running. This consisted of a slight but progressive increase in the distance between the hindlimbs that was first detectable at 7 months of age.

In this study we examine gait phenotypes in a mouse model knocked-in for a spastin ATPase missense mutation, encoding the murine equivalent (N384K) of the human disease-associated N386K missense mutation [8]. Murine and human spastin ATPase domains are identical, and the N386K mutation affects a residue in the entirely conserved (from humans to plants) canonical Walker A motif, which forms the phosphate binding loop (P-loop) of the ATPase domain [18]. Structural studies on Drosophila and human spastin have confirmed that the N386 residue forms a critical part of the P-loop of the nucleotide binding region [18, 22]. Mutations in P-loop residues affect nucleotide binding or hydrolysis, and consistent with this, the N386K mutation almost completely blocks ATP hydrolysis but appears to allow ATP binding [23]. As in previous spastin-HSP models, axonal swellings developed in primary neurons cultured from homozygous mutant mice. We used a motorized treadmill based digital analysis system (DigiGait) to characterize and quantify gait metrics, which revealed several subtle but significant abnormalities of gait parameters in homozygous mutant mice. Our results highlight the utility of this approach in sensitively detecting gait abnormality in models of HSP.

## Materials and Methods

### Knock-in mouse generation and breeding

**Fig 1:**
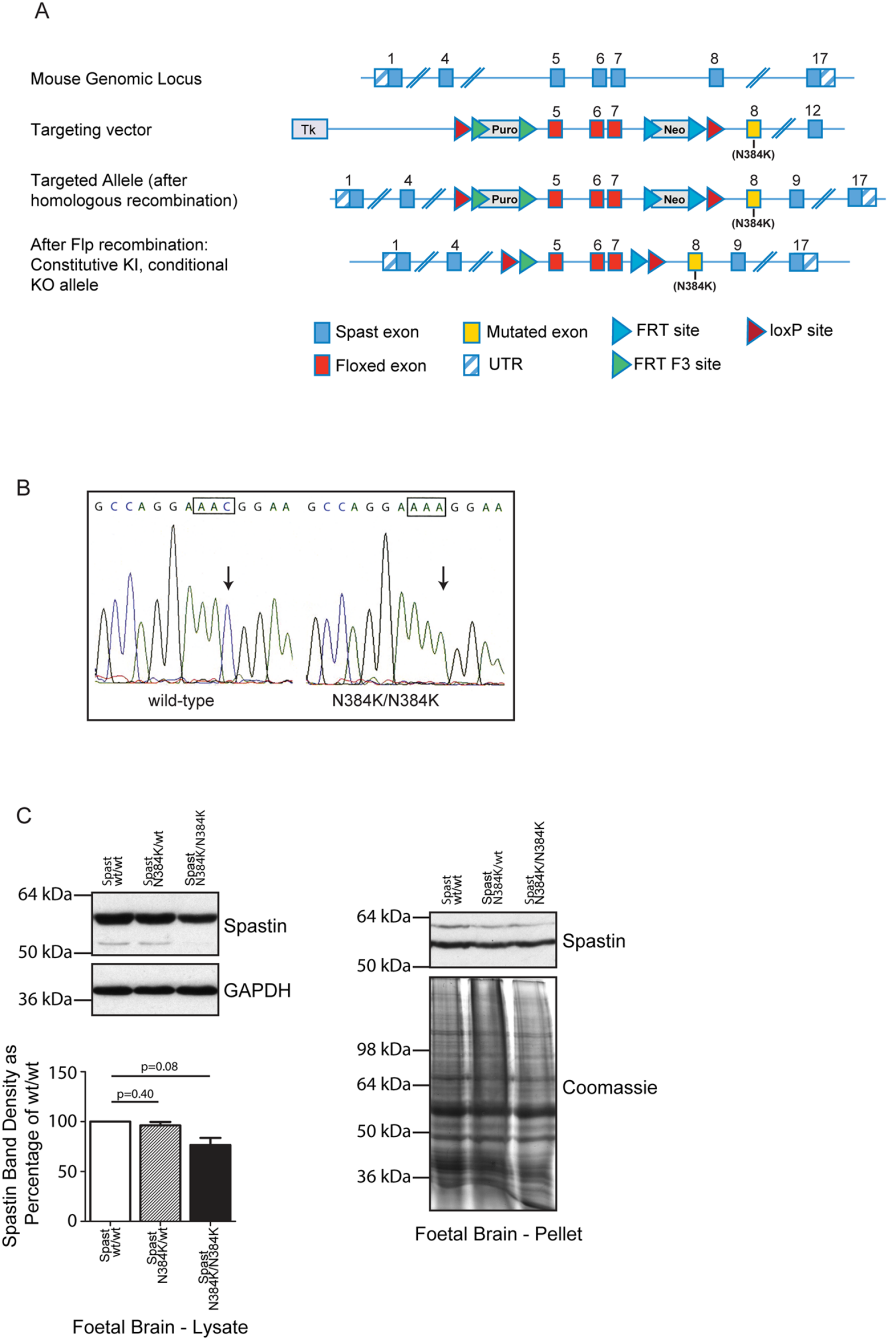
Spastin^N384K/N384K^ knock-in mouse generation and validation. A) Schematic diagram of strategy used to generate spastin^N384K^ allele. Numbering refers to spastin exons. Puro = puromycin resistance cassette, Neo = neomycin resistance cassette, Flp = flippase, FRT = flippase recognition target, UTR = untranslated region. B) DNA sequencing of PCR product generated using primers designed to amplify fragment of spastin exon 8 from genomic DNA of a wild-type mouse and spastin^N384K/N384K^ littermate. C) Immunoblotting versus spastin of lysate or pellet fraction from foetal brain tissue (E17), from mice of the genotypes indicated. The histogram shows corresponding densitometry of the spastin bands in the lysate (n = 3 per genotype, p-values generated by paired t-tests). GAPDH immunoblotting or Coomassie staining serve to verify equal loading of lanes. Size bars indicate molecular weight in kD.

A schematic diagram of the targeting strategy is shown in Fig 1A. We had generated (at Taconic Artemis) mice knocked-in for a mutation in exon 8 (Spast c.1152c<a (p.Asn384Lys), NM_001162870.1, CCDS50181.1) encoding an N384K amino acid substitution, on a C57BL/6 genetic background. Briefly, exons 5–8 of the *spast* locus were replaced by a positive selection cassette containing the mutation. This cassette contained two positive selection markers (neomycin and puromycin resistance) flanked by flippase (Flp) recognition target F3 or FRT sites, in introns 4 and 7 respectively. It also incorporated loxP sites in introns 4 and 7, providing the possibility of future deletion of these exons by crossing with Cre-recombinase expressing mice, to generate a spastin loss-of-function model. The targeting vector was generated using BAC clones from the C57BL/6J RPCI-23 BAC library and transfected into TaconicArtemis C57BL/6N Tac ES cell line. ES clones were analyzed by Southern Blotting to confirm correct recombination and single integration, according to standard procedures. Homologous recombinant clones were used to generate chimeric animals by injection into C57BL/6 blastocysts. Highly chimeric mice were bred to C57BL/6 Flp-Deleter mice, to remove the selective markers. Germline transmission was identified by the presence of black, strain C57BL/6 offspring (G1) and was confirmed by PCR of ear biopsies using the following primer pairs:

2201_31: AAACTGTTCCCAAGGCATCC and 2201_32: GTGTCAGTGTGCATAAGTCATGG, which detect the presence or absence of the loxP site in intron 4,

2084_29: GTCATAGCTGTAACCAACTTCTG and 2084_30: ACCAAACACATGCGAGTGAGG, which amplify an exon 8 sequence containing the mutation. The presence of the mutation was confirmed by sequencing (Source BioScience) (Fig 1B).

Mice were maintained in accordance with United Kingdom and European Union regulations. Animal work for this study was approved by the University of Cambridge Ethical Review Committee and was carried out under project licenses (80/2304 and 70/7888) granted by the United Kingdom Home Office under the Animals (Scientific Procedures) Act 1986. The University of Cambridge is a designated establishment for breeding and for scientific procedures under the Animals (Scientific Procedures) Act 1986.

Mice were housed under temperature-controlled and specific-pathogen-free conditions, with a 12-hour light/dark cycle and free access to drinking water and food. They were maintained on a C57BL/6 background. We interbred heterozygous mice to generate wild-type (termed spast^wt/wt^), heterozygous (termed spastin^N384K/wt^) and homozygous (termed spastin^N384K/N384K^) mice. The cohort of mice analysed in the behavioural studies was made up of groups of spast^wt/wt^, spast^N384K/wt^ and spastin^N384K/N384K^ littermates. Embryonic or day old mice used to generate primary neurons were sacrificed by decapitation, adult mice were sacrificed by cervical dislocation.

### Antibodies

The following antibodies were used: Mouse monoclonal anti-MAP2 (abcam ab11268), mouse monoclonal anti-acetlylated tubulin (Sigma-Aldrich T7541)), rabbit polyclonal anti-spastin (spastin86-340, raised in house to amino acids 86–340 of spastin [24, 25]), rabbit monoclonal anti-GAPDH (Cell Signalling 2118, clone 14C10), mouse monoclonal anti-Tau-5 antibody (Abcam ab80579).

### Generating tissue lysates and western blotting

Cortex from mouse brain was homogenized in ice cold lysis buffer (25mM HEPES pH 7.4, 1mM EDTA, 150mM NaCl, 0.5% TX100, protease inhibitors) using a pellet pestle (Sigma-Aldrich). Homogenate was centrifuged at 5000rpm for 15 min and supernatant centrifuged at 49000rpm for 1 hour. Protein concentration was quantified using BCA protein assay (Pierce) and total spastin levels were analysed in 30μg of lysate protein using SDS-PAGE. Pellet fractions were resuspended in 300μl sample buffer and 15μl was analyzed using SDS-PAGE, with equal lane loading confirmed by Coomassie staining. Gels were blotted onto PVDF membrane and blocked in 5% non-fat dried milk in TBS-Tween. Membrane was incubated with primary antibodies and HRP-conjugated secondary antibodies in blocking buffer and HRP-signal detected with SuperSignal ^®^ West Pico Chemiluminescent Substrate using X-Ray film (Fuji Medical). Immunoblot bands were quantified using ImageJ software.

### Primary Neuron Culture

Cortical neurons were obtained from E17 mouse embryos or day old pups and cultured on Poly-D-Lysine coated glass coverslips in Neurobasal medium (Gibco/Invitrogen) with B27 supplement (Gibco/Invitrogen) and L-glutamine (Sigma-Aldrich). After seven days in culture neurons were fixed in 3.7% formaldehyde and processed for immunofluorescence microscopy.

### Immunofluorescence microscopy

To quantify axonal swellings, formaldehyde fixed neurons on coverslips were permeabilised with 0.1% TX100 in PBS and blocked in 5% fetal calf serum in PBS for 1 hour. Coverslips were incubated with primary antibodies and Alexa Fluor^®^ conjugated secondary antibodies in blocking buffer and mounted onto glass slides using Prolong^®^ Gold antifade reagent with DAPI (Molecular Probes). Neurons were visualized using a Zeiss Axioimager Z2 Wide-field upright microscope and axonal swellings were quantified per 250 nuclei. Axonal swellings were identified as acetylated tubulin positive, MAP2 negative regions on a single axon which were greater than twice the diameter of the axon.

### Tissue genotyping

Genomic DNA was purified from mouse ear biopsies using DNeasy^®^Blood and Tissue kit (Qiagen). PCR was performed using primer pairs 2201_31 and 2201_32 on an Applied Biosystems GeneAmp^®^9700 PCR system. PCR products were analysed on a 1.5% agarose gel and genotype was identified by the presence or absence of bands at 235bp (wt/wt), 235 and 427bp (wt/N384K), 427bp (N384K/N384K).

### Modified Shirpa Analysis

The following behaviours chosen from the modified Shirpa analysis were recorded for each mouse at 1 year old: body position, tremor, palpebral closure, coat appearance, presence of whiskers, presence of defecation, transfer arousal, locomotor activity in arena by measuring number of squares entered with all 4 feet in 3 seconds, tail elevation, touch escape, positional passivity, trunk curl, limb grasping and evidence of biting [26].

### DigiGait analysis

DigiGait equipment was purchased from Mouse Specifics, Inc (Boston) and analysis was carried out as previously described [27]. Briefly, each mouse was placed on a motor-driven treadmill with a transparent treadmill belt and imaged from beneath with a high-speed digital video camera. A minimum of three seconds of movie is required for digigait analysis, and mice that could not run at the chosen speed for that duration were deemed to have failed to complete the test. Colour images were converted to their binary matrix equivalents and the areas of the moving paws relative to the belt and camera were calculated throughout each stride. This was used to generate a dynamic gait signal of the paw placement relative to the treadmill belt and camera. Each limb’s gait signal was used to calculate the stride duration (time duration of one complete stride for the paw under analysis). This was broken down into subcomponents of stance duration (the time duration when the paw is in contact with treadmill) and swing duration (the time duration when the paw is above the walking surface and not in contact with the belt). Stance duration further comprises the braking phase (time duration from initial paw contact with the treadmill to maximum paw contact) and propulsion duration (time duration from maximum paw contact to lifting from treadmill). Stride width was defined as the perpendicular distance between the centroids of each set of axial paws during peak stance. Gait symmetry was measured as the ratio of forelimb stepping frequency to hind limb stepping frequency.

### Statistical Analysis

Statistical analyses were carried out using GraphPad Prism 5.01 for Windows (GraphPad Software, San Diego) statistical software. Specific statistical tests used are described in the relevant figure or table captions. Briefly, for behavioral analysis, one-way (to analyse effects of a single variable, i.e. genotype) or two-way (to analyse effects of two variables, i.e. gender and genotype) ANOVA was performed to compare effects across 3 groups (spast^wt/wt^, spast^N384K/wt^ or spast^N384K/N384K^). In certain cases ANOVA was supplemented with Bonferroni post-test for comparison of individual pairs of genotypes. Fischer’s exact test was used to analyse the contingency table of digigait success or failure by genotype. Differences in spastin immunoblot band densitometry were assessed by paired t-tests. For p-values, * = <0.05, ** = <0.01, *** = <0.001, **** = < 0.0001. Error bars in all graphs represent mean ± standard error of the mean (S.E.M.).

## Results

### Generation of spastin knock-in mice

We used a gene targeting approach to generate a mouse model on a C57BL/6 background, which was knocked in for a mutation (1152c<a) in exon 8 of murine spastin. This mutation encodes an N384K missense change in the spastin protein, corresponding to the human disease-causing missense mutation N386K [8]. The presence of the mutation was confirmed by sequencing (Fig 1B). Spastin protein was expressed in brain tissue from spastin^wt/wt^, spastin^N384K/wt^ and spastin^N384K/N384K^ animals (Fig 1C).

### Axonal swellings in primary neurons from knock-in mice

**Fig 2:**
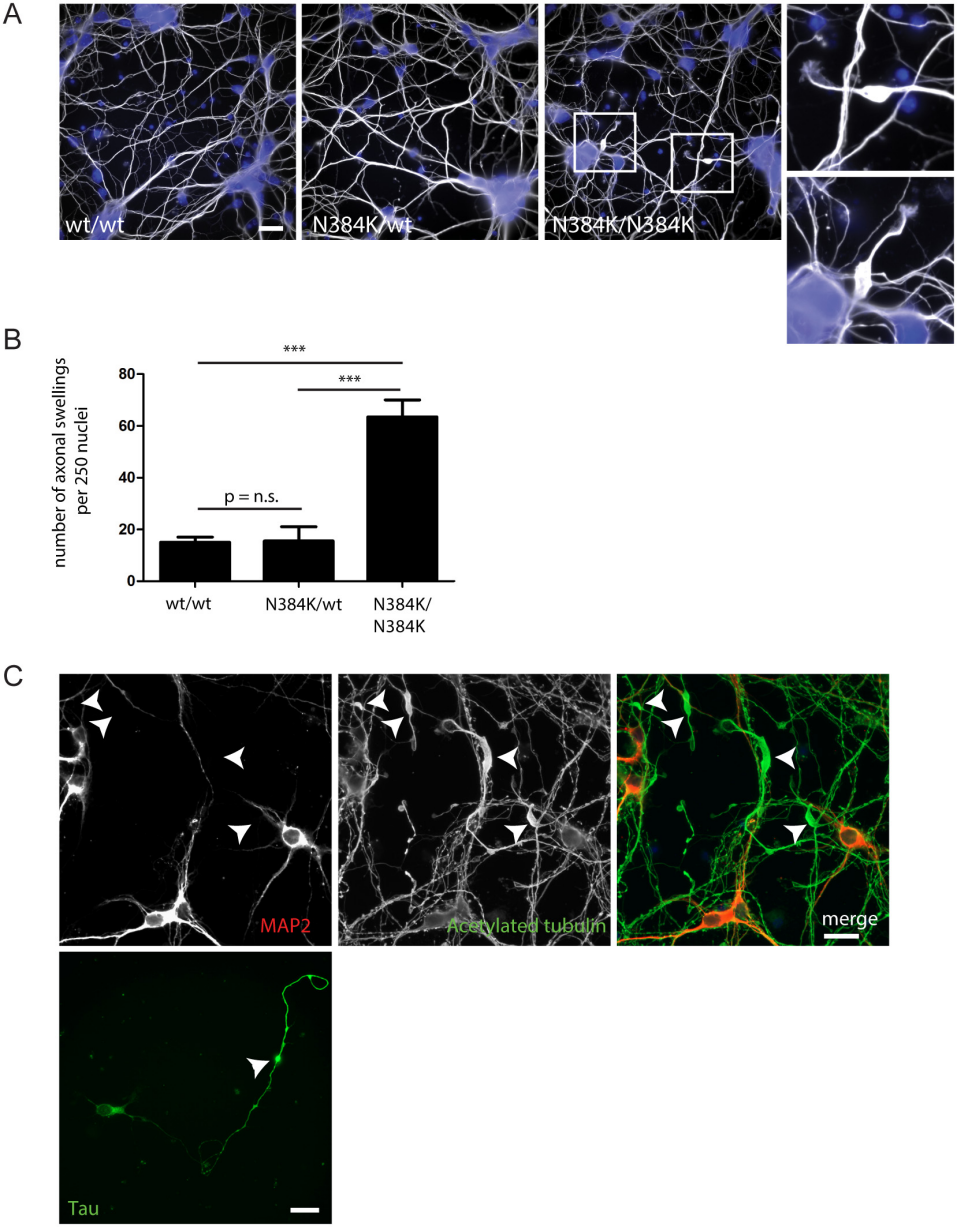
Spastin^N384K/N384K^ mice develop axonal swellings. A) Day 7 *in vitro* primary cortical neurons from animals with the genotypes indicated labeled with antibody to acetylated tubulin. DAPI staining (blue) was used to label nuclei. Examples of swellings seen in spastin^N384K/N384K^ neurons are shown in the boxed areas, which are magnified in the right hand panels. B) Primary cortical neurons were labeled as in A) and the number of swellings per 250 nuclei was quantified in animals with the genotypes shown (n = 3 repeats per genotype). Mean number of swellings per 250 nuclei ± S.E.M.: spast^wt/wt^ = 16.0 ± 1.53, spast^N384K/wt^ = 16.3 ± 3.28 and for spast^N384K/wt^ 62 ± 4.04. P-values were generated by 1-way ANOVA with Bonferroni post-test for comparison of individual pairs of genotypes. C) Day 7 *in vitro* primary cortical neurons from spastin^N384K/N384K^ mice were labeled with the antibodies indicated. Swellings (arrowheads) were always MAP2 negative or tau positive, indicating that they were present in axons. Scale bars = 20μm.

In light of the primary neuron axonal swelling phenotype observed in previous spastin mouse models, we examined whether similar swellings were present in primary neurons derived from spastin^N384K/N384K^ mice. We saw prominent neurite swellings in spastin^N384K/N384K^ primary neurons and the affected neurites never labeled for the somatodendritic marker MAP2 but labeled with the axonal marker tau, confirming them as axons (Fig 2A–2C). Spastin^N384K/wt^ neurons did not develop significantly more axonal swellings than wild-type littermates (Fig 2B). These results demonstrate the presence of a cell-autonomous neuronal pathology, and are consistent with findings in other spastin mouse models. Furthermore, they demonstrate no obvious dominant negative or gain-of-function effects of the mutant protein in heterozygous neurons.

### Gait abnormality in spastin^N384K/N384K^ mice

**Fig 3:**
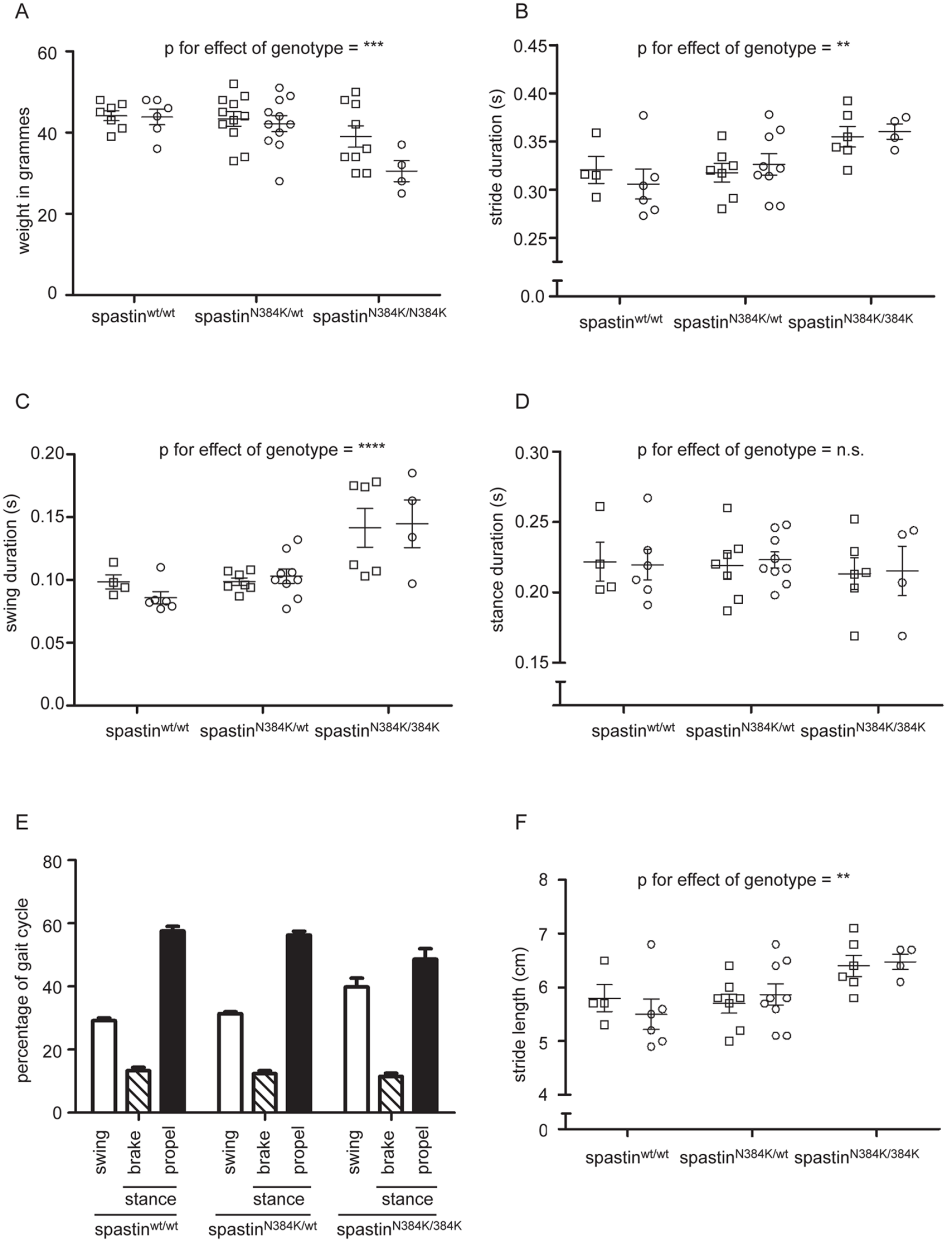
Weight and key left hindlimb gait parameters in spastin^N384K/N384K^ mice versus littermate controls at 1 year old. Plots show values for each animal tested with the genotypes indicated. Squares = male animals, circles = females. A) weight in grammes, B) stride duration, C) swing phase duration, D) stance phase duration. E) Histogram showing the mean percentage of time that tested animals with the genotypes indicated spent in each phase of gait. F) Stride length in animals with the genotypes shown. Error bars = mean ± S.E.M. Numerical p-values for corresponding statistical tests using two-way ANOVA for the effect of gender and genotype on gait parameters are shown in Table 2. To facilitate interpretation, p-values for genotype are indicated on the plots by * p<0.05, ** p<0.01, *** p<0.001, **** p<0.0001.

Heterozygous mutant mice bred normally, and both heterozygous and homozygous mutants showed no obvious defects in size or morphology. However, at 1 year old, spastin^N384K/N384K^ mice were significantly lighter than heterozygous or wild-type mice (Fig 3A). We characterized the locomotor phenotype of a cohort of 1 year old male and female littermate spastin^N384K/wt^ and spastin^N384K/N384K^ animals, as well as littermate spast^wt/wt^ mice. No obvious phenotypic differences between the different genotypes were detected on modified primary SHIRPA examination. We also examined the maximum running speed of the animals at 1 year using an accelerating treadmill, with treadmill speeds increasing by 1cm/s at 10s intervals, beginning from 5cm/s. Again, we found no significant effect of genotype.

We next used the DigiGait analysis system to quantify gait parameters while the mice were running on a treadmill. This system images the ventral side of mice as they move on a motorized transparent treadmill belt and quantifies spatial and temporal gait indices [27]. These include the duration for a complete stride (stride duration), as well as individual components of the stride, such as the duration of swing and stance phases (see Methods section for a full description of the gait parameters analysed). As many rodent gait parameters are influenced by running speed [28], we used a constant speed of 18cm/s. This running speed was chosen to optimize the number of animals able to complete the testing, as at higher speeds increasing numbers of mice of all genotypes were not able to keep up with the treadmill, most likely because of their relatively advanced age. Even so, some animals were unable to run at 18cm/s and so were excluded from analysis, but the proportion excluded did not significantly differ by genotype (Table 1). Using 2-way ANOVA, we found significant effects of genotype, but not of gender in a number of basic hind limb gait metrics (Table 2 and Fig 3). In particular, the spastin^N384K/N384K^ mice had a significantly lengthened mean stride duration, and the increase was almost entirely due to an elongation of the swing phase of gait (Table 2, Fig 3B–3D and S1–S4 Movies). These changes altered the profile of a typical step in the spastin^N384K/N384K^ mice, compared to the other two genotypes (Fig 3E). Mean stride length was also significantly increased in the spastin^N384K/N384K^ mice, consistent with the need to maintain a constant speed with a slower stride (Table 2 and Fig 3F).

**Table 1:** Numbers of animals in cohort unable to complete DigiGait analysis at 1 year. p-value for association between genotype and completion/failure of test was not significant (Fischer’s exact test).

Genotype	Completed DigiGait assessment at 18cm/s	Failed DigiGait assessment at 18cm/s
spast^wt/wt^	10	3
spastin^N384K/wt^	16	6
spastin^N384K/N384K^	10	3

**Table 2:** Weight and Quantified gait parameters in spast^wt/wt^, spastin^N384K/wt^, spastin^N384K/N384K^ mice at 1 year old. Values are shown for the left hind limb unless otherwise stated; very similar results were obtained for the right hind limb. p-values for the effect of gender and genotype were calculated using two-way ANOVA. To correct for multiple testing, the alpha value for significance was calculated by dividing 0.05 by 8, using a highly conservative assumption of 8 independent tests (many of the tests are in reality related), to give α = 0.00625. spast^wt/wt^ n = 10 (4 male, 6 female), spastin^N384K/w^t n = 16 (7 male, 9 female), spastin^N384K/N384K^ n = 10 (6 male, 4 female).

Parameter	Mean ± S.E.M. for: spast^wt/wt^ spastin^N384K/wt^ spastin^N384K/N384K^	p-value for effect of Gender	p-value for effect of genotype
**Weight (g)**	44.0 ± 1.06	0.08	0.0008
	42.8 ± 1.30		
	36.4 ± 2.23		
**Mean stride duration (s)**	0.31 ± 0.011	0.96	0.005
	0.32 ± 0.007		
	0.36 ± 0.007		
**Mean swing phase duration (s)**	0.09 ± 0.004	0.83	< 0.0001
	0.10 ± 0.003		
	0.14 ± 0.01		
**Mean stance phase duration (s)**	0.22 ± 0.01	0.88	0.79
	0.22 ± 0.02		
	0.21 ± 0.01		
**Mean braking duration in stance phase (s)**	0.04 ± 0.0005	0.33	0.98
	0.04 ± 0.002		
	0.04 ± 0.003		
**Mean propulsion duration in stance phase (s)**	0.18 ± 0.0005	0.61	0.82
	0.18 ± 0.005		
	0.17 ± 0.004		
**Mean stride length (cm)**	5.7 ± 0.15	0.92	0.006
	5.8 ± 0.08		
	6.4 ± 0.04		
**Mean hindlimb stance width (cm)**	2.8 ± 0.11	0.09	0.06
	2.7 ± 0.03		
	3.0 ± 0.19		
**Mean gait symmetry ratio (1 = entirely symmetrical)**	1.01 ± 0.01	0.42	0.64
	1.00 ± 0.006		
	1.00 ± 0.005		

To analyse whether there was evidence that the N384K mutation acted in a dominant fashion, we next analysed the key gait metrics that were influenced by genotype, specifically to determine whether heterozygous mutant animals differed significantly from homozygous mutant or wild-type animals. As the 2-way ANOVA analysis demonstrated no effect of gender on these parameters, we pooled data from males and females, and compared the effect of each genotype against the other two. While we saw significant differences between wild-type and spastin^N384K/N384K^ animals, and between spastin^N384K/wt^ and spastin^N384K/N384K^, we saw no significant difference between spastin^wt/wt^ and spastin^N384K/wt^ mice (Table 3). We thus saw no dominant effect of the mutation on the gait phenotype.

**Table 3:** Comparison of genotypes for selected gait parameters. P-values generated using one-way ANOVA with Bonferroni post-test for multiple comparison of individual pairs of genotypes.

Gait Parameter	p-value for spast^wt/wt^ versus spastinN384K/N384K	p-value for spastinN384K/wt versus spastinN384K/N384K	p-value for spast^wt/wt^ versus spastinN384K/wt
**Mean stride duration (s)**	<0.01	<0.05	n.s.
**Mean swing phase duration (s)**	<0.001	<0.001	n.s.
**Mean stride length (cm)**	<0.01	<0.05	n.s.

### Abnormal gait parameters can be detected at 4 months old

**Fig 4:**
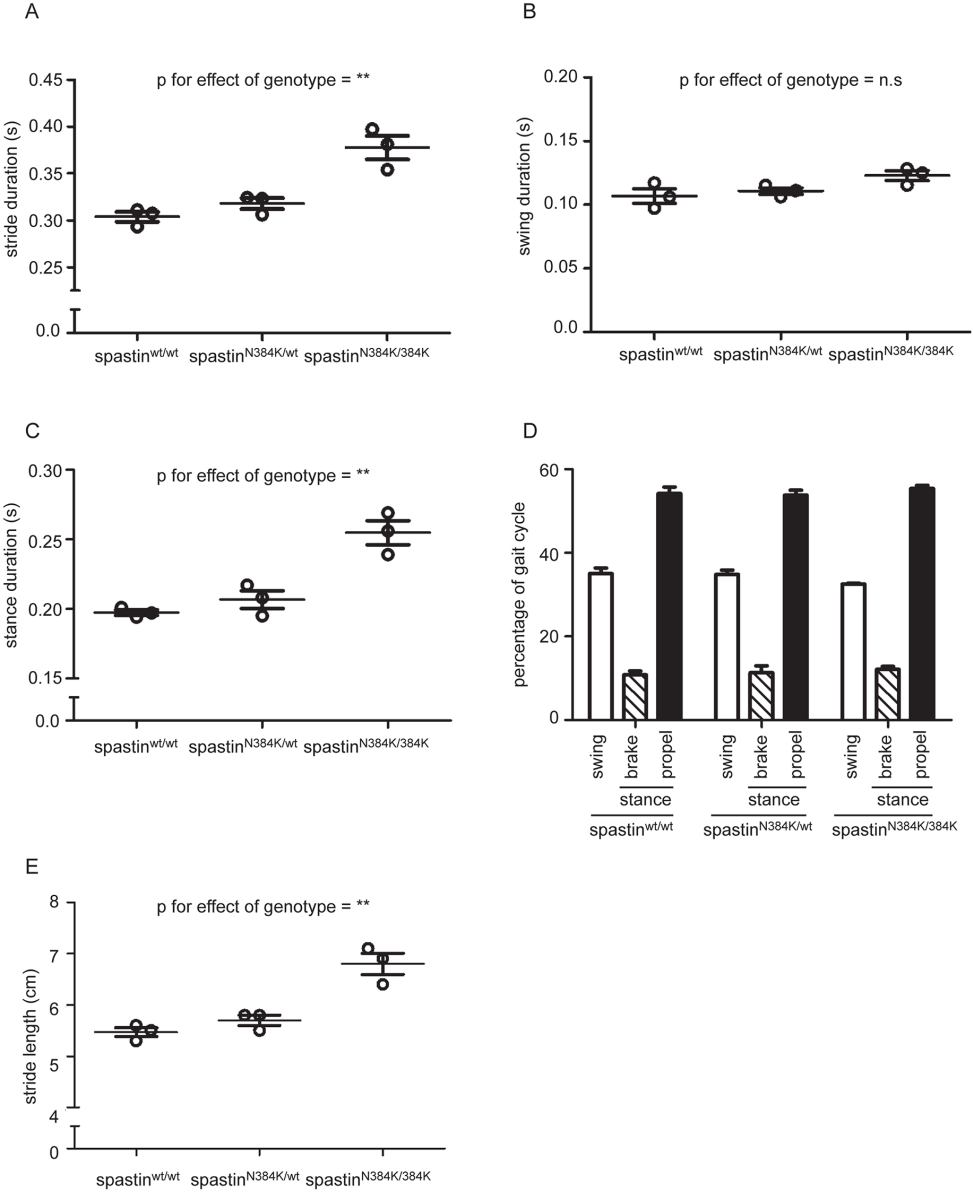
Key left hindlimb gait parameters in spastin^N384K/N384K^ mice versus littermate controls at 4 months old. Plots show values for each animal tested with the genotypes indicated. A) stride duration, B) swing phase duration, C) stance phase duration. D) Histogram showing the mean percentage of time that tested animals with the genotypes indicated spent in each phase of gait. E) Stride length in animals with the genotypes shown. Numerical values for corresponding statistical tests using one-way ANOVA for the effect of genotype on gait parameters are shown in Table 4. To facilitate interpretation, p-values for genotype are indicated on the plots by * p<0.05, ** p<0.01, *** p<0.001, **** p<0.0001. Error bars = mean ± S.E.M

Having characterised gait parameters in the 1-year old cohort of spastin-HSP mice, we tested whether gait abnormalities could be detected at a younger age by analyzing gait metrics in a separate cohort of mice at 4 months old. Of note, in wild-type mice at this age, key gait parameters including stride duration, stance duration and swing duration were very similar to those previously obtained by Digigait in a group of 3 month old C57BL/6 mice running at a similar speed [29]. However, we found a significant increase in stride duration in spastin^N384K/N384K^ mice versus littermate controls (Table 4 and Fig 4A). This was not restricted to the swing phase of gait, as stance phase duration was significantly increased (Table 4 and Fig 4B and 4C). Thus overall, the gait profile was not affected by genotype (Fig 4D). Finally, as with the older mice, there was a significant increase in stride length in spastin^N384K/N384K^ compared to wild-type and heterozygous littermates, consistent with the need to run at the same speed with a slower stride duration (Table 4 and Fig 4E). We also analysed, as described above for the 1 year old mice, whether there was any dominant effect of the mutation in heterozygous mice, but found no significant difference between wild-type and heterozygous mice for any gait parameter analysed.

**Table 4:** Quantified gait parameters in spast^wt/wt^, spastin^N384K/wt^, spastin^N384K/N384K^ mice at 4 months old. Values are shown for the left hind limb unless otherwise stated. p-values for the effect of genotype were calculated using one-way ANOVA. To correct for multiple testing, the alpha value for significance was calculated by dividing 0.05 by 8, using a highly conservative assumption of 8 independent tests (many of the tests are in reality related), to give α = 0.00625. N = 3 mice in each group.

Gait Parameter	Mean ± S.E.M. for: spast^wt/wt^ spastin^N384K/wt^ spastin^N384K/N384K^	p-value for effect of genotype
**Mean stride duration (s)**	0.30 ± 0.005	0.002
	0.32 ± 0.006	
	0.38 ± 0.01	
**Mean swing phase duration (s)**	0.11 ± 0.006	0.088
	0.11 ± 0.003	
	0.12 ± 0.004	
**Mean stance phase duration (s)**	0.20 ± 0.01	0.0014
	0.21 ± 0.02	
	0.25 ± 0.01	
**Mean braking duration in stance phase (s)**	0.033 ± 0.003	0.16
	0.036 ± 0.006	
	0.046 ± 0.009	
**Mean propulsion duration in stance phase (s)**	0.16 ± 0.002	0.0003
	0.17 ± 0.002	
	0.21 ± 0.006	
**Mean stride length (cm)**	5.5 ± 0.09	0.0012
	5.7 ± 0.1	
	6.8 ± 0.2	
**Mean hindlimb stance width (cm)**	2.6 ± 0.07	0.52
	3.0 ± 0.29	
	2.9 ± 0.18	
**Mean gait symmetry ratio (1 = entirely symmetrical)**	1.02 ± 0.02	0.28
	1.04 ± 0.01	
	1.08 ± 0.04	

## Discussion

We generated a novel genetically modified mouse knocked-in for a disease-associated missense mutation that results in expression of an ATPase-defective form of spastin, thus modelling a class of spastin mutations that has not previously been analysed in mammalian *in vivo* systems. In contrast to our model, in which enzymatically dead spastin is expressed, previous mouse models have involved loss of the spastin protein, caused either by a deletion of exons 5–7, or defective splicing of exon 7 [20, 21]. Nevertheless, like these earlier models, in our model homozygous mutant animals had axonal swellings in cortical neuronal cultures (which contain the target cells of HSP) and a gait abnormality. The gait abnormality was evident on quantitative gait testing, but was subtle as it was not detected on modified Shirpa testing. Many gait metrics change with running speed, and so to allow direct comparison of metrics between animals, gait was assessed at a constant speed [28]. At both 4 months and one year old we found that the spastin^N384K/N384K^ mice had a symmetrical gait, but that they had a significantly longer stride duration than control mice. Step length was also increased in the spastin^N384K/N384K^ mice, and this is consistent with findings in one of the knock-out models [20].

The identification of quantifiable gait abnormalities as early as 4 months old in the spastin^N384K/N384K^ mice will be useful in assessing the therapeutic response to future pharmacological agents designed to treat HSP. Indeed, some gait metrics differed by genotype significantly in 4 month old mice but not in 1 year old mice (e.g. stance duration). With the caveat that we examined relatively few 4 month old animals, so that we may by chance have under-estimated the inter-animal variability in gait metrics at this age, we speculate that this may be because degenerative changes affecting gait parameters may be more prevalent in the older animals, so increasing the variability of results (compare for example the variability in wild-type mouse results for stride duration in Fig 3B versus Fig 4A). This would tend to obscure subtle differences between control and homozygous knock-in mice.

In contrast to the situation in humans, where heterozygous spastin mutations are sufficient to cause HSP, we saw no frank axonal swellings or convincing locomotor phenotype in mice heterozygous for the spastin N384K-expressing allele, although in the 4 month old animals there was a trend towards several gait metrics for heterozygous animals being intermediate between wild-type and homozygous mutant values. While mice may require increased dosage of mutant alleles to drive development of neurodegenerative diseases within their short (compared to humans) lifespan [30], significant effects on axonal swelling or gait phenotypes might have been expected in heterozygous mice if the mutation acted via a dominant negative or gain-of-function pathological mechanism. Thus while not formally excluding them from playing a role in human patients, our results provide no strong support for these mechanisms of disease acting in our mouse model.

## Conclusions

We have developed a novel mouse model expressing an ATPase-defective form of spastin. Homozygous mutant mice develop a subtle but reliably detectable gait abnormality. Our results highlight the utility of quantitative gait analysis using treadmill systems in identifying gait abnormalities in models of hereditary spastic paraplegia.

## Supporting Information

S1 File Minimal data set. (XLSX)

S1 Movie Representative example of ventral imaging produced by the Digigait system of a 1 year old wild type mouse running on a transparent treadmill at 18cm/s. (MOV)

S2 Movie Representative example of ventral imaging produced by the Digigait system of 1 year old spastin^N384K/wt^ mouse running on a transparent treadmill at 18cm/s. (MOV)

S3 Movie Representative example of ventral imaging produced by the Digigait system of 1 year old spastin^N384K/N384K^ mouse running on a transparent treadmill at 18cm/s. The mouse shown has a relatively significant gait abnormality.
(MOV)

S4 Movie Representative example of ventral imaging produced by the Digigait system of 1 year old spastin^N384K/N384K^ mouse running on a transparent treadmill at 18cm/s. The mouse shown has a relatively subtle gait abnormality.
(MOV)
